# ROS-mediated EB1 phosphorylation through Akt/GSK3β pathway: implication in cancer cell response to microtubule-targeting agents

**DOI:** 10.18632/oncotarget.1982

**Published:** 2014-05-18

**Authors:** Marion Le Grand, Amandine Rovini, Veronique Bourgarel-Rey, Stephane Honore, Sonia Bastonero, Diane Braguer, Manon Carre

**Affiliations:** ^1^ Aix Marseille Université, Inserm, CRO2 UMR_S 911, Marseille, France; ^2^ APHM, Hopital Timone, Marseille, France; ^3^ Department of Neurology, Mayo Clinic, Rochester, MN, USA

**Keywords:** EB1 phosphorylation, microtubule dynamics, mitochondrial ROS, chemotherapy, Akt/GSK3β pathway

## Abstract

Microtubule-targeting agents (MTAs) are largely administered in adults and children cancers. Better deciphering their mechanism of action is of prime importance to develop more convenient therapy strategies. Here, we addressed the question of how reactive oxygen species (ROS) generation by mitochondria can be necessary for MTA efficacy. We showed for the first time that EB1 associates with microtubules in a phosphorylation-dependent manner, under control of ROS. By using phospho-defective mutants, we further characterized the Serine 155 residue as critical for EB1 accumulation at microtubule plus-ends, and both cancer cell migration and proliferation. Phosphorylation of EB1 on the Threonine 166 residue triggered opposite effects, and was identified as a requisite molecular switch in MTA activities. We then showed that GSK3β activation was responsible for MTA-triggered EB1 phosphorylation, resulting from ROS-mediated inhibition of upstream Akt. We thus disclosed here a novel pathway by which generation of mitochondrial ROS modulates microtubule dynamics through phosphorylation of EB1, improving our fundamental knowledge about this oncogenic protein, and pointing out the need to re-examine the current dogma of microtubule targeting by MTAs. The present work also provides a strong mechanistic rational to the promising therapeutic strategies that currently combine MTAs with anti-Akt targeted therapies.

## INTRODUCTION

Mitochondria are dynamic organelles that are organized in cells as a network closely connected to microtubules. They constitute a major source of Reactive Oxygen Species (ROS), which can form as a natural byproduct of the normal metabolism of oxygen through oxidative phosphorylation. ROS mainly consist in superoxide anion radical (O_2.-_), hydroxyl radical (OH.) and hydrogen peroxide (H_2_O_2_). Among them, H_2_O_2_ acts as a second messenger for many cellular processes such as migration, proliferation and differentiation [1-3]. While a basal level of ROS is essential for cell viability, a mitochondria-governed oxidative stress has been linked to cell death induction through the intrinsic apoptotic pathway [4-5] and to mitochondrial network fragmentation that contributes to cell dysfunctions [6-7]. This growing set of data paved the way for new strategies that take into account the functional capacity of mitochondria to produce ROS in cancer cells, and that aim at interfering with mitochondrial energetics to improve cancer treatment [8-9].

Microtubule-Targeting agents (MTAs) are a broad group of anticancer drugs that are currently administered in a large range of indications in adults and children. It is well established that MTAs activate the intrinsic apoptotic pathway, and especially mitochondria membrane permeabilization and cytochrome c release [extensively reviewed in 10-11]. MTA anti-mitochondrial activities can result from a modulation of Bcl-2 family members' expression levels [12-14] or from a direct targeting of mitochondrial membranes [15-16]. MTAs are largely described to primary alter microtubule dynamic instability, resulting in suppression of both essential mitotic and interphase processes, such as cell proliferation, polarization and migration [17-19]. A growing set of data shows that MTA efficacy depends on microtubule plus end-tracking proteins (+TIPs), which control multiple aspects of microtubule dynamic instability [20-21]. The End-Binding protein 1 (EB1) forms comet-like accumulation at the growing microtubule plus-ends – by recognizing the GTP-cap and tubulin conformational intermediate states induced by GTP hydrolysis [22-23] – and plays a central role in assembly of +TIPs complexes [24-25]. The physiological functions of EB1 have been largely reviewed in a variety of microtubule-mediated cellular activities, including migration and cell division [26-27]. In addition, EB1 is overexpressed in several cancers [28-29] and we recently showed that this overexpression selectively sensitized glioblastoma to *in vitro* and *in vivo* MTA treatment (Berges *et al*., submitted). Our previous works also reported that alteration of EB1 accumulation at microtubule plus-ends was a fundamental event in MTA mechanism of action [30-32]. The intracellular machinery responsible for regulation of EB1 binding at microtubules is still under extensive investigations and discussions. Recent studies reported phosphorylation of EB3, another member of the EB family [33-34], increasing the range of +TIPs subjected to this post-translational modification [35-36]. While a phosphorylation of EB1 homologues (Bim1p and Mal3) has been pointed out in budding and fission yeasts [37-38], this process has not been yet identified in mammalian cells.

To date, the role of mitochondrial ROS in microtubule dynamics regulation has never been evaluated. However, in 2010, Smith [39] showed that an exogenous H_2_O_2_-mediated oxidative stress led to EB1 release from microtubule plus-ends, in cervix adenocarcinoma cells and cardiomyocytes from mice. Here, we aimed at characterizing the influence of mitochondrial ROS on microtubule dynamics and functions, and its involvement in cell response to MTA chemotherapy. We first showed that mitochondrial ROS overproduction was determining for MTA-mediated alteration of EB1 accumulation and cell response to drug cytotoxic and anti-migratory activities, in cancer cells of various tissue origins. We then highlighted that EB1 was subjected to phosphorylation, which regulates its ability to bind to microtubules. We further characterized Serine 155 and Threonine 166 as the potential phosphorylated residues, which exerted distinct effects on EB1 accumulation and microtubule dynamics. GSK3β activation, that resulted from ROS-mediated inhibition of Akt, was lastly identified as responsible for MTA-triggered EB1 phosphorylation. Altogether, our results revealed a novel signaling pathway between mitochondria and microtubules, which could have high potential for therapeutic strategies and better understanding of cellular processes.

## RESULTS

### Mitochondrial ROS overproduction governs MTA cytotoxic and anti-migratory activities

**Figure 1:**
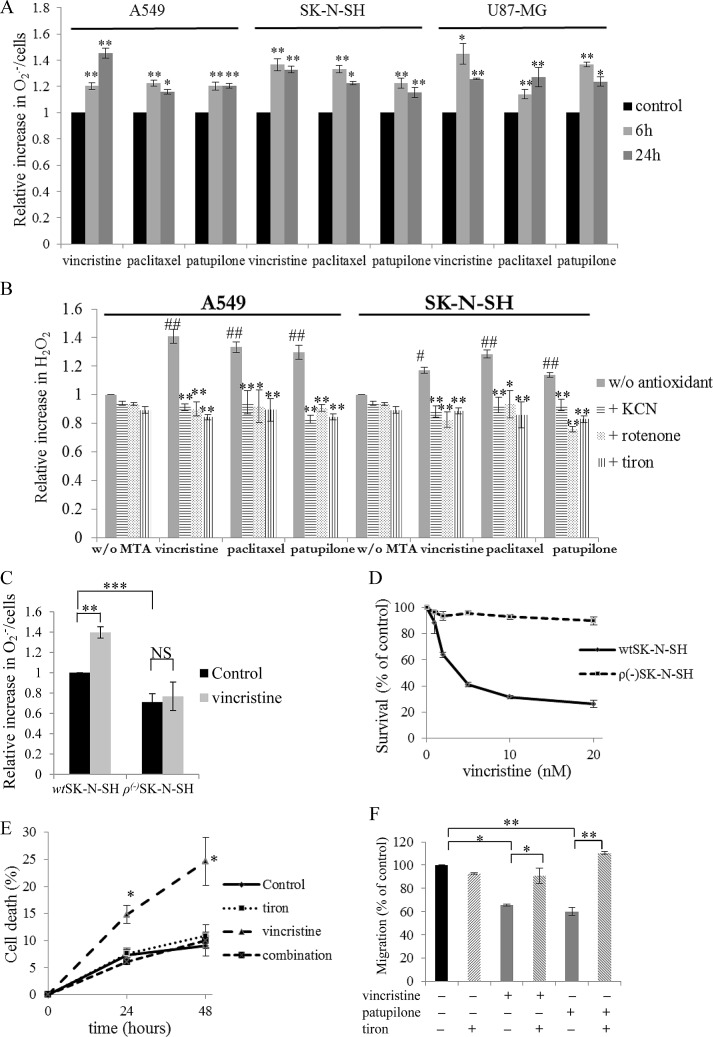
Mitochondrial ROS overproduction governs MTA cytotoxic and anti-migratory activities (A) Relative production of superoxides by WST-1 assay in A549, SK-N-SH and U87-MG cells during vincristine, paclitaxel or patupilone treatment at 2 nM at 6 and 24 h. (B) Relative generation of hydrogen peroxide by the H_2_DCFDA fluorescence test in A549 and SK-N-SH cells incubated with 2 nM of vincristine, paclitaxel or patupilone, ROS inhibitors/scavengers or their combination for 6 h (MTAs are compared to control, while combinations are compared to MTAs alone). (C) Relative production of superoxides measured by WST-1 assay in *wt*SK-N-SH and *ρ*^(-)^SK-N-SH cells treated with 2 nM vincristine for 6 h. (D) *wt*SK-N-SH and *ρ*^(-)^SK-N-SH cell survival revealed by the MTT test after a 72 h-exposition to vincristine. (E) Detection of apoptosis by flow cytometry analysis of annexin-V and propidium iodide staining in A549 cells treated with vincristine (2nM), tiron or their combination for 24 and 48 h. (F) Measurement of transwell migration in U87-MG cells in the presence of 2 nM of vincristine or patupilone, tiron or their combination for 6 h. Data are presented as mean ± S.E.M. Student's *t*-test *, *p*<0.05; **, *p*<0.01.

To evaluate the ability of MTAs to modulate mitochondrial ROS generation, several human cancer cell lines were used, originating from non-small cell lung carcinoma (A549), neuroblastoma (SK-N-SH), and glioblastoma (U87-MG). As shown in Fig.1A, 2nM vincristine, paclitaxel and patupilone (*i.e.* representative of the main MTA sub-classes used in the clinic at concentrations around IC_50_ *in vitro*) promoted O_2_^.-^ production in the three cell lines. For example, in SK-N-SH cells, O_2_^.-^ production by mitochondria was increased by 36.6 ± 4.5 % after a 6 h-treatment with vincristine, and was maintained to a similar level after 24 h (32.9 ± 2.7 %; p<0.01). In agreement, H_2_O_2_ intracellular levels were significantly increased by MTAs, whatever the cancer cell line evaluated (Fig.1B and Supplementary Fig 1A). The anticancer drugs were then combined to KCN, rotenone or tiron. These co-incubations resulted in the entire suppression of O_2_^.-^ and H_2_O_2_ overproduction leading to ROS basal level return whatever the inhibitor used (Fig.1B and Supplementary Fig 1B). To definitely ascertain the mitochondrial origin of the MTA-triggered ROS overproduction, we developed mtDNA-mutated *ρ^(-)^*SK-N-SH cells, that were characterized by a decrease in ROS production from mitochondria (– 29 ± 3 %; *p*<0.001; Fig.1C). Unlike parental *wt*SK-N-SH cells, *ρ^(-)^*SK-N-SH cells were unable to overproduce O_2_^.-^ after a 6 h-treatment with vincristine (Fig.1C). Altogether, these data clearly show that MTAs share the capacity to selectively enhance ROS generation from mitochondria in different human cancer cell lines.

To specify the role of ROS overproduction in MTA activity, we first measured the drug effects on *wt* and *ρ^(-)^*SK-N-SH cell survival. As shown in Fig.1D and Supplementary Fig 1C, *ρ^(-)^*SK-N-SH cells were highly resistant to a 72 h-treatment with vincristine or patupilone. Even concentrations that caused up to a 90 % decrease in *wt*SK-N-SH cell survival remained ineffective in *ρ^(-)^*SK-N-SH cells. Consistently, the time-dependent induction of A549 cell death by vincristine was suppressed when combined with tiron (Fig.1E and Supplementary Fig 1D). Transwell® assays further revealed that ROS overproduction was also essential for the anti-migratory effects of MTAs. These experiments have been performed in U87-MG cells, since glioblastoma are among the most invasive cancers. As shown in Fig.1F, vincristine and patupilone treatment significantly decreased cell migration, by 34 ± 6 % (*p*<0.05) and 40 ± 3 % (*p*<0.01) respectively. However, when combined with tiron, MTAs did not display anti-migratory properties anymore. Inhibition of mitochondrial ROS overproduction is thus a source of cell resistance to both cytotoxic and anti-migratory effects of treatment.

### ROS overproduction by MTAs is responsible for inhibition of EB1 accumulation at microtubule plus-ends

**Figure 2:**
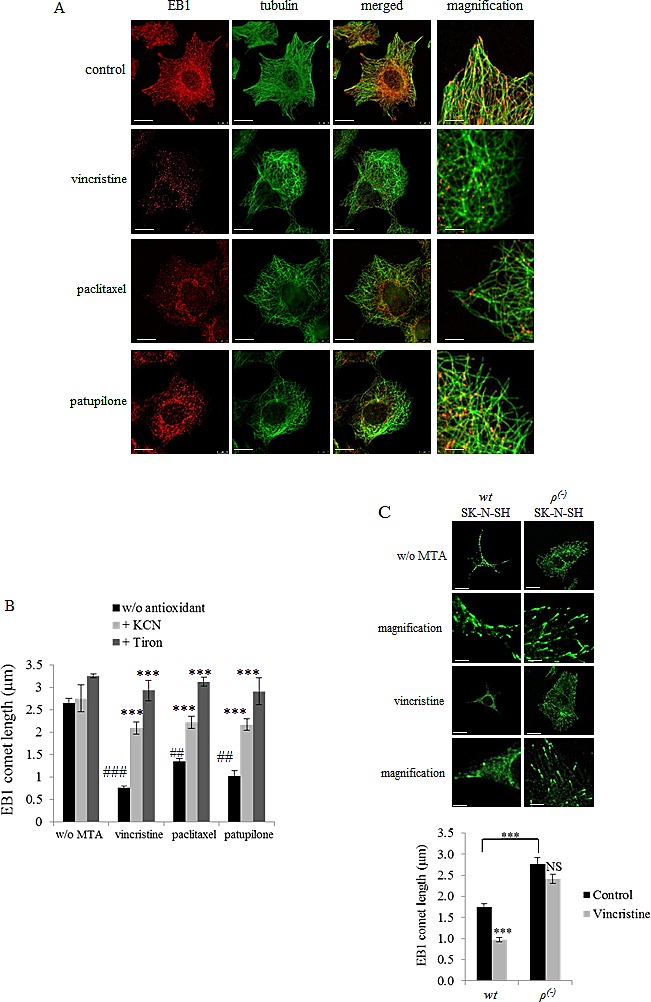
MTA-mediated ROS overproduction inhibits EB1 accumulation at microtubule plus-ends (A) Double indirect immunofluorescence staining of EB1 (red) and α–tubulin (green) in A549 cells incubated with 2 nM of vincristine, paclitaxel or patupilone for 6 h. Scale bars, 20μm. Magnified images: Scale bars, 5μm. (B) Quantification of EB1 comet length in A549 cells treated with MTAs and/or antioxidants for 6 h. Scale bars, 2.5μm. (MTAs are compared to control while combinations are compared to MTAs alone). (C) Immunofluorescence staining of EB1 and quantification of EB1 comet length in *wt*SK-N-SH and *ρ(-)*SK-N-SH under 6 h-treatment of vincristine at 2nM. Scale bars, 10μm. Magnified images: Scale bars, 2.5μm. Data are presented as mean ± S.E.M. Student's t-test **, *p*<0.01; ***, *p*<0.001.

MTAs are known to affect cell proliferation and migration *via* inhibition of EB1 accumulation at microtubule plus-ends and alteration of microtubule dynamics instability. Here, we intended to understand whether mitochondrial ROS are be involved in such processes caused by MTAs. Confocal microscopy revealed a typical pattern of EB1 with comet-like structures at the plus-ends of microtubules in A549 control cells (Fig.2A, control panels). As expected, treatment with MTAs for 6 h significantly inhibited EB1 accumulation at microtubule plus-ends (Fig.2A). Measurement of EB1 comets yielded a length from 2.7 ± 0.1 µm in control cells to 1.4 ± 0.1, 0.8 ± 0.1 and 1.0 ± 0.1 µm respectively in cells incubated with paclitaxel, vincristine and patupilone (*p*<0.01) (Fig.2B). It is noteworthy that EB1 expression level was not modulated by MTA treatment (Supplementary Fig 2A), strongly suggesting that EB1 proteins were delocalized at the microtubule plus-ends. We then evaluated the impact of KCN and tiron on EB1 localization in A549 cells. Interestingly, their simultaneous combination with MTAs prevented drug-induced EB1 delocalization (Fig.2B). For instance, the impact of vincristine on EB1 comet length was limited to 30 % when combined with KCN and totally suppressed by tiron. These results were similarly obtained in U87-MG and SK-N-SH cells (Supplementary Fig 2B, 2C and data not shown). It should be noticed that treatment with tiron alone increased the length of EB1-decorated plus-end of microtubules by 23 % in A549 cells (Fig.2B) and by 13 % in U87-MG cells (Supplementary Fig 2C), strengthening the link between intracellular ROS levels and EB1 ability to accumulate at microtubule plus-ends.

To further confirm these unexpected results, we measured EB1 comet length in *wt* and *ρ^(-)^*SK-N-SH cells. Consistently with the 29 ± 3 % decrease in basal mitochondrial O_2_^.-^ levels (Fig.1C), comet length was increased in *ρ^(-)^*SK-N-SH cells to 2.8 ± 0.2 µm (Fig.2C), as compared with the parental *wt* cells (1.7 ± 0.1 µm; *p*<0.001). We also quantified the median number of EB1 comets per cell, that increased from 160 ± 10 in *wt*SK-N-SH cells to more than 270 ± 22 in *ρ^(-)^* cells suggesting that number of growth microtubules increased (data not shown). Vincristine treatment (for 6 h), which was highly effective in *wt*SK-N-SH cells, caused a complete disappearance of EB1 accumulation at microtubule plus-ends in 88 % of the cell population (data not shown). In cells that still contained EB1 comets, vincristine decreased their length from 1.7 ± 0.1 µm to 1.0 ± 0.1 µm (*p*<0.001; Fig. 2C). Interestingly, in *ρ^(-)^*SK-N-SH cells, vincristine did neither alter EB1 comet length (2.8 ± 0.2 to 2.4 ± 0.1 µm; *p*>0.05) nor comet number (data not shown) in the majority of cell population (92 % of cells; data not shown). These data definitely involve ROS generation by mitochondria in regulation of EB1 accumulation at microtubule plus-ends.

### Phosphorylation of EB1 regulates its accumulation at microtubule plus-ends, microtubule dynamics and microtubule-governed cell functions

**Figure 3:**
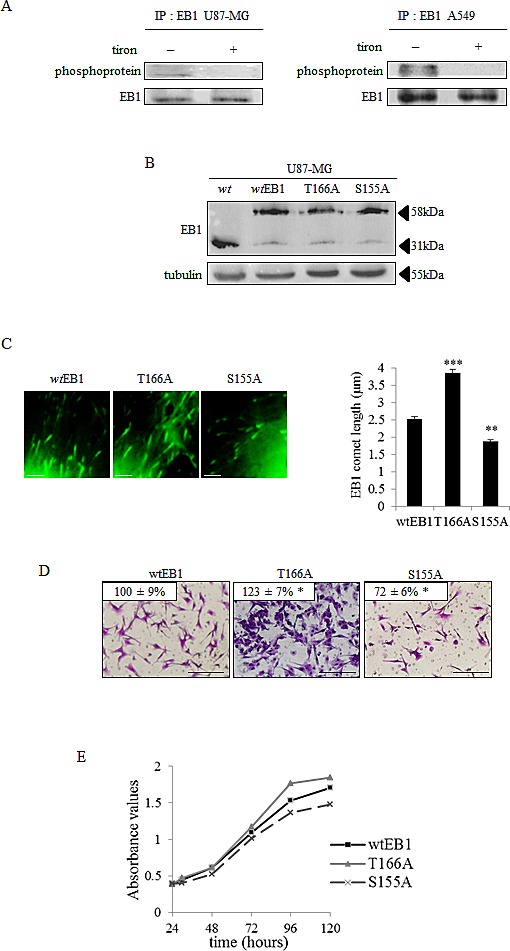
Phosphorylation of EB1 modulates its microtubule binding, microtubule dynamics and the microtubule-governed cell functions (A) Immunoprecipitation (IP) was performed in U87-MG and A549 cell lysates with anti-EB1 monoclonal antibody and the precipitates were analysed by western blot probed with anti-phosphoprotein total and anti-EB1 antibodies. (B) Western Blot analysis of EB1 expression in *wt*EB1-GFP, EB1 T166A-GFP, EB1 S155A-GFP transfected U87-MG cells. Parental U87-MG wild type (wt) was used as a negative control and α–tubulin was used as loading control. (C) Representative images and quantification of EB1 comet length in U87-MG cells transfected with *wt*EB1-GFP, EB1 T166A-GFP and EB1 S155A-GFP. Scale bars, 2.5μm. (D) Measurement of transwell migration in EB1 T166A and EB1 S155A U87-MG transfected cells, expressed as a percentage of transmigrated cells in *wt*EB1 cells. Panel shows representative images of the lower surface filter taken with an Olympus microscope. Scale bars, 200µm. (E) Proliferation assay in *wt*EB1, EB1 T166A and EB1 S155A U87-MG transfected cells. Data are presented as mean ± S.E.M. Student's t-test *, *p*<0.05; **, *p*<0.01; ***, *p*<0.001.

A growing body of evidence has recently demonstrated that +TIPs, including EB3, can be subjected to phosphorylation. To analyze whether such a process could be responsible for changes in EB1 accumulation at microtubule plus-ends, we performed EB1 immunoprecipitation associated with anti-phosphoprotein antibody. For the first time, we found a phosphorylated form of EB1 in U87-MG cell lysates (Fig.3A, left panel). We also revealed that EB1 phosphorylation was suppressed by the O_2_^.-^ scavenger tiron. This ROS-dependent post-translational modification of EB1 was then confirmed in A549 cells (Fig.3A, right panel). Since tiron also increased EB1 comet length (see Fig.2B and Supplementary Fig 2C), one can reasonably hypothesize that phosphorylation may control EB1 ability to form comets at microtubule plus-ends. Using PhosphoSite, we thus generated constructs encoding for phospho-defective human EB1 protein, *via* substitution of threonine 166 or serine 155 residues by an alanine residue. We first ascertained that endogenous EB1 expression was repressed in favor of exogenous EB1-GFP in the stably transfected U87-MG cells with the EB1 T166A-GFP, EB1 S155A-GFP, and non-mutated *wt*EB1-GFP constructs (Fig.3B). We also ensured that the length of *wt*EB1-GFP comets was similar to the endogenous EB1 comets stained by immunofluorescence (2.5 ± 0.2 µm *vs*. 2.2 ± 0.1 µm; *p*>0.05; Fig 3C and supplemental Fig 2C). We then measured that comet length was significantly increased to 3.9 ± 0.2 µm in T166A mutant cells, as compared with comets in *wt*EB1 cells that were 2.5 ± 0.2 µm (*p*<0.001) (Fig.3C and Table 1). Consistently with this, we showed by live imaging fluorescence microscopy that the T166A mutation of EB1 significantly increased microtubule growth rate (15.9 ± 0.6 µm.min^−1^ *vs.* 10.6 ± 0.4 µm.min^−1^ in *wt*EB1 cells) and reduced catastrophe frequency (0.7 ± 0.1 µm^−1^ *vs.* 1.0 ± 0.1 µm^−1^ in *wt*EB1 cells) (Table 2 and supplementary videos 1 and 2). The time of existence of EB1 binding sites at microtubule plus-ends, termed “decoration time”, was not different in the T166A-EB1 cells and in the *wt*EB1 cells (14.7 ± 0.2 s and 14.2 ± 0.1 s respectively), since EB1 comet length and microtubule growth rate increased in a similar way (Table 2). The S155A mutation differently interfered with the microtubule system. EB1 comet length was conversely decreased to 1.9 ± 0.1 µm in S155A mutant cells (*p*<0.01; Fig.3C and Table 1), microtubule growth rate was only increased by 18 % (p<0.05) and the catastrophe frequency was not significantly modified (p>0.05) as compared with *wt*EB1 cells (Table 2 and supplementary videos 1 and 3). In addition, decoration time of microtubule plus-ends by EB1 S155A was decreased by 40 %, indicating that regulation of EB1 binding by phosphorylation on the S155 residue is distinct to what occurs when EB1 is phosphorylated on the T166 residue.

**Table 1:** Measurement of EB1 comet length in wtEB1-GFP, EB1 T155A-GFP and EB1 S166A-GFP transfected U87-MG cells treated with vehicle (control) or vincristine 2 nM for 6 h Data are presented as mean ± S.E.M. Student's t-test ****, p<0.001; NS, p>0.05

EB1 comet length (µm)	control	vincristine	vincristine vs control
wtEB1	2.5 ± 0.1	1.0 ± 0.1	− 60 % ***
T166A	3.9 ± 0.1	3.4 ± 0.1	− 13 % NS
S155A	1.9 ± 0.1	1.0 ± 0.1	− 47 % ***

**Table 2:** Measurement of microtubule dynamics parameters by live microscopy of EB1 at microtubule plus-ends in wtEB1-GFP, EB1 T155A-GFP and EB1 S166A-GFP transfected U87-MG cells exposed to vehicle (control) or vincristine 2 nM for 6 h EB1 comets were tracked over time to measure microtubule growth rate and catastrophe frequency. Data are presented as mean ± S.E.M. Student's t-test *, p<0.05; **, p<0.01; ***, p<0.001; NS, p>0.05

	variables	control	vincristine	vincristine vs control
	Growth rate (µm.min-1)	10.6 ± 0.4	7.5 ± 0.5	− 30 % ***
wtEB1	Catastrophe frequency (µm-1)	1.0 ± 0.1	1.7 ± 0.1	+ 65 % ***
	Decoration time (s)	14.2 ± 0.1	8.0 ± 0.3	− 56 % ***
	Growth rate (µm.min-1)	15.9 ± 0.6	15.9 ± 0.7	< 1 % NS
T166A	Catastrophe frequency (µm-1)	0.7 ± 0.1	0.9 ± 0.1	+ 26 % *
	Decoration time (s)	14.7 ± 0.2	12.8 ± 0.3	− 13 % NS
	Growth rate (µm.min-1)	12.5 ± 0.5	8.5 ± 0.7	− 31 % ***
S155A	Catastrophe frequency (µm-1)	0.9 ± 0.1	1.5 ± 0.2	+ 56 % **
	Decoration time (s)	9.0 ± 0.1	7.0 ± 0.2	− 23 % *

Lastly, we explored the relevance of EB1 phosphorylation in microtubule-governed cell functions. T166A mutation led to slight increases in cell migration (+ 23 ± 7 %; *p*<0.05) and cell proliferation (up to + 15 % at 96h) as compared to *wt*EB1 cells (Fig.3D and 3E). On the opposite, S155A mutation resulted in the decrease of the U87-MG cells migration (– 28 ± 6 %; *p*<0.05), as well as cell proliferation (– 11 % at 96h). Thus, depending on the residue targeted, EB1 phosphorylation results in quite opposite effects on microtubule dynamics and microtubule-governed cancer cell functions.

### ROS-mediated EB1 phosphorylation is involved in MTA activities

**Figure 4:**
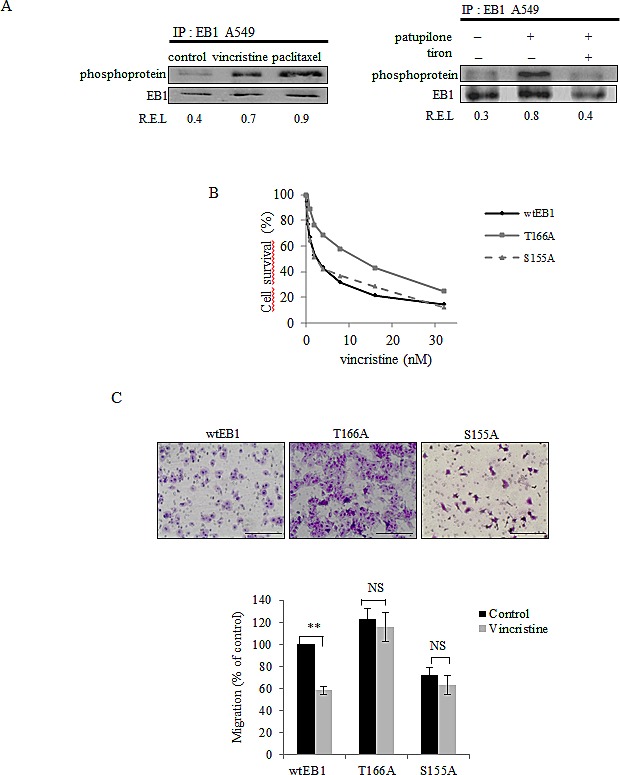
ROS-mediated EB1 phosphorylation is involved in MTA activities (A) A549 cells were treated for 6 h with 2 nM of vincristine, paclitaxel and patupilone or combined with tiron, and lysed. Immunoprecipitation was performed as described in legend of Figure 3A. (B) Survival of *wt*EB1, EB1 T166A and EB1 S155A transfected U87-MG cells exposed to vincristine, measured by the MTT test. (C) Migration of U87-MG transfected cells (see details in Figures 3D legend) after a 6 h-treatment with vincristine 2 nM. Data are presented as mean ± S.E.M. Student's t-test NS, *p*>0.05; **, *p*<0.05.

To investigate whether EB1 phosphorylation play a role in MTA activity, we performed EB1 immunoprecipitation from MTA-treated A549 cells (for 6 h). We showed that 6 h-treatment of vincristine, paclitaxel and patupilone increased EB1 phosphorylation 1.7, 2.5 and 3.2 times, respectively (Fig.4A, left and right panels). We revealed thus that patupilone-mediated EB1 phosphorylation was governed by ROS, since it was suppressed by a simultaneous combination with tiron (Fig.4A, right panel). These data were validated in U87-MG cells exposed to vincristine (Supplementary Fig 3).

In agreement with results obtained in U87-MG cells, vincristine decreased EB1 comet length by 60 % in *wt*EB1-GFP transfected U87-MG cells (Table 1). Further, treatment also inhibited microtubule dynamics, *via* both a decrease in microtubule growth rate (– 30 %) and a huge increase in catastrophe frequency (+ 65 %) in *wt*EB1 cells (Table 2). Importantly, vincristine became almost completely ineffective in reducing comet length at microtubule plus-ends in EB1 T166A-GFP cells (Table 1). In these mutant cells, the MTA-mediated increase in catastrophe frequency was limited to 26 %, while the decrease in microtubule growth rate was totally prevented by the T166A substitution (Table 2). On the opposite, the S155A mutation did not protect EB1 comet and microtubule dynamics from vincristine-induced damages (Tables 1 and 2).

We then explored if changes in EB1 phosphorylation influence MTA activities. As shown in Fig.4B, T166A cells were significantly less sensitive to cytotoxic effects of vincristine (for 72 h), with an IC_50_ value 4.4 fold-increased as compared to *wt*EB1 cells (9.2 ± 0.2 nM and 2.1 ± 0.4 nM respectively; *p*<0.05). Moreover, T166A mutation of EB1 inhibited the anti-migratory activity of vincristine (for 6 h) (Fig.4C). In EB1 S155A mutated cells, unlike in T166A cells, vincristine cytotoxicity was maintained (IC_50_ value 2.1 ± 0.2 nM; Fig.4B), and vincristine anti-migratory effects were similar as those measured in *wt*EB1 cells (– 37 % and – 42 % respectively).

Collectively, our results revealed that inhibition of EB1 T166 residue phosphorylation results in cancer cell insensitivity to MTA anti-microtubule activities, as well as resistance to MTA cytotoxic and anti-migratory properties. Conversely, MTAs are still effective in S155A mutant cells.

### The Akt/GSK3β pathway governs EB1 phosphorylation in MTA treated cells

**Figure 5:**
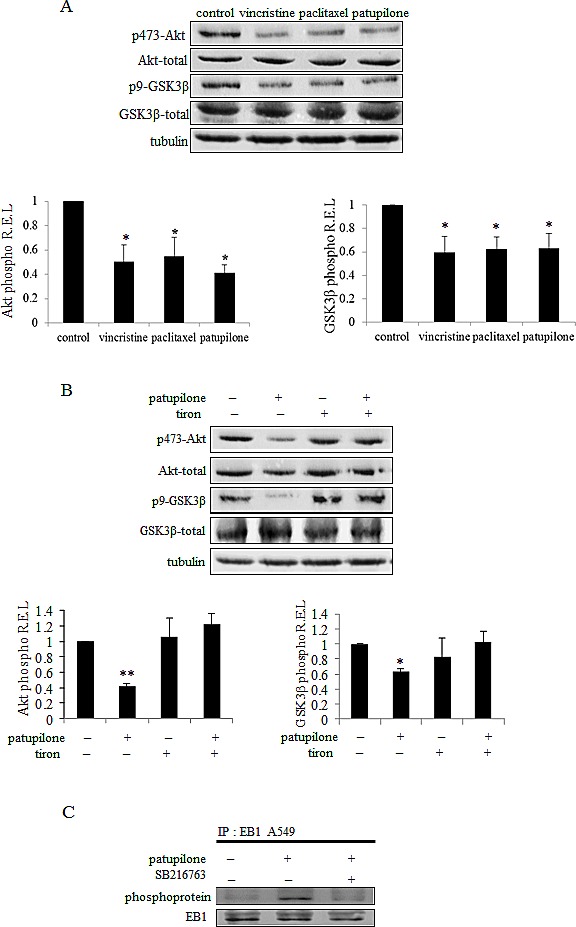
ROS-mediated Akt/GSK3β pathway governs EB1 phosphorylation under MTA treatment (A) Western blot analysis of expression and activity of Akt and GSK3β under 6 h-treatment with 2 nM of vincristine, paclitaxel or patupilone in A549 cells. Quantification of western blot bands, expressed as phospho/total protein ratio; α–tubulin was used as loading control. (B) Same experiment in cells exposed to patupilone, tiron or their combination. (C) A549 cells were treated for 6 h with 2 nM of vincristine or combined with SB216763. Immunoprecipitation was performed as described in Figure 3A legend. Data are presented as mean ± S.E.M. Student's t-test *, *p*<0.05; **, *p*<0.01.

**Figure 6:**
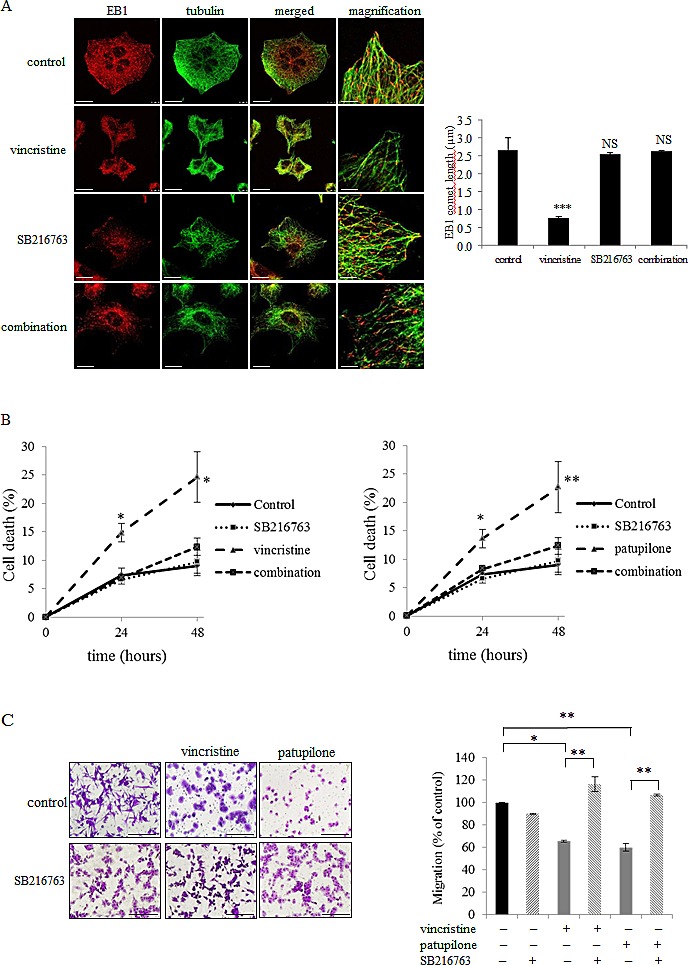
GSK3β activation governs EB1 accumulation at microtubule plus-ends and MTA activities (A) Double indirect immunofluorescence staining of EB1 (red) and α–tubulin (green) and quantification of EB1 comet length in A549 cells incubated with 2 nM of vincristine, SB216763 or their combination for 6 h. Scale bars, 20μm. Magnified images: Scale bars, 5μm. (B) Detection of apoptosis in A549 cells by flow cytometry analysis of annexin-V and propidium iodide staining under 2 nM of patupilone, SB216763 or their combination for 6 h. (C) Measurement of transwell migration in U87-MG cells under 6 h-treatment of vincristine or patupilone 2 nM, SB216763 or their combination. See Figure 3D legend for details. Data are presented as mean ± S.E.M. Student's t-test *, *p*<0.05; **, *p*<0.01; ***, *p*<0.001.

We next sought to identify the molecular actors responsible for EB1 phosphorylation downstream of mitochondrial ROS generation. It has been reported that Microtubule-Associated Proteins (MAP) can be phosphorylated by GSK3β [40]. To investigate the involvement of the Akt/GSK3β signaling pathway in MTA-mediated EB1 phosphorylation, we first assessed the expression of phosphorylated S473 Akt, as well as S9 GSK3β. As shown in Fig.5A, 6 h-treatment with vincristine, paclitaxel or patupilone similarly led to a decrease by 50 % in the phosphorylated active form of Akt. Concomitantly, we showed a decrease in GSK3β phosphorylation (40 %, 37 % and 36 % respectively for the three MTAs), which is correlated with its activation. To determine whether ROS overproduction was involved in Akt inhibition, MTA-treated cells were simultaneously incubated with tiron. Interestingly, the O_2_^.-^ scavenger restored initial phospho-Akt and phospho-GSK3β levels (Fig.5B and Supplementary Fig 4A), indicating that MTA-triggered ROS overproduction was responsible for Akt inactivation and, consequently, GSK3β activation. Then, to determine the role of GSK3β in EB1 phosphorylation, A549 cells were treated with MTA combined with the GSK3β specific inhibitor SB216763 during 6 h. As shown in Fig. 5C, SB216763 suppressed patupilone-mediated phosphorylation of EB1, clearly pointing out that GSK3β is required to achieve EB1 phosphorylation. Consistently, SB216763 prevented EB1 accumulation at microtubule plus-ends in vincristine-treated A549 cells (0.8 ± 0.1 µm and 2.6 ± 0.9 µm for vincristine and the combination respectively; *p*>0.05) (Fig.6A). Same results were obtained in U87-MG cells (Supplementary Fig 4B). Lastly, we showed that GSK3β was involved in both pro-apoptotic and anti-migratory activities of MTAs. For instance, SB216763 reduced 2.0 and 1.9 times A549 cell death induced by a 48 h-treatment with vincristine and patupilone respectively (*p*<0.01; Fig. 6B). Likewise, we showed that combination of MTAs with SB216763 resulted in a total suppression of the anti-migratory effect of vincristine and patupilone (Fig. 6C). Of note, we also analyzed AMPK activity, that appeared not to be involved in MTA activities (data not shown). Altogether, our results highlight a pivotal role for the ROS-modulated Akt/GSK3β pathway that, *via* EB1 phosphorylation and accumulation to microtubule plus-ends, governs MTA efficacy.

## DISCUSSION

Understanding anticancer drug mechanism of action is of prime importance, not only for deciphering resistance processes but also for developing more convenient cancer therapy strategies. Here, we disclosed a novel mechanism by which generation of mitochondrial ROS suppresses microtubule dynamics, through Akt/GSK3β-mediated phosphorylation of EB1. Importantly, we identified this signaling bridge between mitochondria and microtubules as responsible for a considerable part of cancer cell response to MTA cytotoxic and anti-migratory activities.

EB1 is a conserved and ubiquitous member of the +TIPs family that regulates the growth and the polymerization of microtubules [41-42]. EB1 represents core element of a dynamic network at the growing microtubule plus-ends and regulate microtubule dynamics through recruitment of others +TIPs [24-25].We previously showed that MTA anti-cancer and anti-angiogenic efficacy correlated with EB1 comet disruption in human neuroblastoma, glioblastoma and endothelial cells [30-32]. Processes underlying regulation of EB proteins binding to microtubule plus-ends have been the object of intensive investigations, and post-translational modifications such as detyronisation /retyrosination or acetylation of the EB1 C-terminal domain have been recently proposed [43-44]. The data currently available also reported phosphorylation of EB3 in endothelial and HeLa cells [33-34]. Phosphorylation of EB1 homologues (Bim1p and Mal3) has been shown in budding and fission yeasts [37-38], but there was still no evidence for such a process in mammalian cells. In the present study, we showed for the first time that EB1 was phosphorylated in human cancer cells of various tissue origins. By using phospho-defective mutants, we further identified Serine 155 and Threonine 166 as the potential residues subjected to phosphorylation. These residues are located in the EB1 linker region, which connects the N-terminal calponin homology domain with the C-terminal domain of EB proteins. The linker region of EB1 tightly contributes to its microtubule binding by promoting the Calponin-Homology domain binding [24 and 45]. Here, we showed that phosphorylation on the S155 residue is required to EB1 accumulation at microtubule plus-ends, and both cancer cell migration and proliferation. Our results are supported by a recent study that linked EB3 stabilization during mitosis to its phosphorylation on S176 by Aurora kinases [46]. Phospho-defective S155 mutant decreased microtubule decoration time by EB1, suggesting that S155 phosphorylation may increase the time of existence or the number of EB1 binding sites at microtubule plus-ends. Further, EB1 is now considered to specifically recognize both the tubulin GTP nucleotide state and the GTP hydrolysis-induced conformational states [22-23]. S155 phosphorylation may thus increase the time of existence of these intermediate forms by inhibiting GTP hydrolysis. The phospho-defective T166A mutant sharp contrasted with the S155A mutant since it promoted EB1 binding in a typical comet-like accumulation and induced microtubule growth through an increase cell proliferation and migration in cancer cells. Interestingly, EB3 phosphorylation on the S162 residue, which is also localized in the linker region, has been similarly identified to be required to destabilize the EB3 dimer and suppress microtubule growth [33]. Lastly, unlike S155 residue, T166 phosphorylation may appear to be required for the decrease of the EB1 decoration time of microtubules by MTAs. We characterized phosphorylation of EB1 on T166 residue as a requisite molecular switch in MTA efficacy, from microtubule dynamics alterations to cytotoxic and anti-migratory consequences.

We identified the serine/threonine kinase GSK3β as responsible for MTA-triggered EB1 phosphorylation and decrease in EB1 accumulation at microtubule plus-ends. EB1 can thus now be included to the class list of +TIP proteins that are substrates of GSK3β and associate with microtubules in a phosphorylation dependent manner [40]. Interestingly, we showed that GSK3β activation was essential for anti-migratory properties of MTA chemotherapy. Besides the well-known role of GSK3β in cell migration *via* APC [47], GSK3β-mediated phosphorylation of EB1 is a likely determining step in the migratory process. In support to this, previous works reported the importance of EB1 to stabilize microtubule plus-ends at cell cortex and target adhesion sites during migration [26, 30]. In the present study, we also showed that GSK3β participated to cancer cell death induced by MTAs. This may be related to the pro-apoptotic properties of GSK3β, through phosphorylation of apoptotic regulators (such as Bax or Mcl-1) [48-49]. GSK3β activation resulted from Akt inactivation in MTA-treated cancer cells. Since the GSK3β selective inhibitor SB216763 only partially protected cells from MTA-induced cytotoxicity, we can easily hypothesize that inactivation of the pro-survival kinase Akt is also involved in the cell death process, by modulating either apoptotic actors or transcription factor activity [50]. Aberrantly high activation of Akt is a characteristic feature of resistance to multiple chemotherapies in a large variety of human cancers [51]. Akt inhibitors are currently evaluated in clinical trials, in combination with conventional cytotoxic drugs [52]. Especially, combination of perifosine or MK-2206 with paclitaxel in ovarian, gastric and melanoma cancers has been recently shown to be a very promising approach [53-55]. In support to this, inhibition of the PI3K/Akt/mTOR pathway by NVP-BEZ235 potentiates effects of vincristine and reduces chemoresistance in *in vitro* and *in vivo* leukemia and sarcoma models [56-57]. By highlighting the key role of the Akt/GSK3β pathway in MTA mechanism of action, our results (i) support the interest for such therapeutic combinations to emphasize response to chemotherapy and overcome drug resistance, and (ii) give for the first time an explanation to their molecular mechanism.

It has become clear that ROS can influence activity of numerous kinases and thereby control cell behavior [58]. Here, we showed that GSK3β activation in MTA-treated cells relies on mitochondrial ROS-mediated inhibition of upstream Akt. Molecular and chemical blockade of ROS overproduction ultimately led to cell insensitivity to both MTA cytotoxic and anti-migratory activities. Furthermore, very preliminary results have shown a deficit in ROS generation induction from mitochondria in multi-drug resistant cells (personal data). A more complex approach than only targeting of microtubules is thus warranted. Accordingly, a direct targeting of mitochondria by MTAs incorporated in novel mitochondriotropic nanocarriers has shown a gain in efficacy [59-61]. While mitochondria silencing has long been accepted as general consensus in cancer cells (Warburg effect), recent studies revealed that this intracellular network is still functional, even in cells that shifted cellular energy metabolism to glycolysis [62]. Hence, a potential therapeutic strategy may consist in the re-activation of mitochondrial oxidative metabolism, including by inhibiting glycolysis [63-64]. Interestingly, the glycolytic phenotype of cancer cells has been reported to be induced by Akt upregulation [65]. A very recent study supports this link between energetic metabolism and Akt pathway, since GSK3β has been reported to potentiate mitochondria biosynthesis stimulation induced by a 2-Desoxy-Glucose-mediated inhibition of glycolysis [66]. Thus, combining MTAs to anti-Akt targeted therapies (as discussed above) might also be efficient through induction of a switch from a glycolytic to an oxidative phenotype in cancer cells.

To conclude, by characterizing a novel phosphorylation-mediated regulation of EB1, the present study improves our fundamental knowledge about this oncogenic protein, which influences processes involved in cancer progression. We also showed the role played by mitochondrial ROS to initiate such a regulatory process of EB1 that leads to MTA efficacy, pointing out the need to reexamine the current dogma of microtubule targeting by MTAs. Lastly, our work provides a strong mechanistic rational to the promising therapeutic strategies that combine MTA-based conventional chemotherapy with anti-Akt targeted therapies.

## MATERIAL AND METHODS

### Cell culture

Human non-small lung carcinoma (A549), neuroblastoma (SK-N-SH) and gliobastoma (U87-MG) cells were obtained from ATCC (Manassas, VA, USA). They were grown in RPMI-1640 medium or in MEM medium supplemented with 1% L-glutamine (Lonza, Levallois-Perret, France), 10 % fetal calf serum (Lonza), 1 % penicillin/streptomycin (Sigma-Aldrich, Saint-Louis, MO, USA). Rho-negative (*ρ^(-)^*) SK-N-SH cells were obtained by incubating parental (*wt*) SK-N-SH cells for 8 weeks with ethidium bromide as previously described [4]. All cell lines were routinely maintained in culture at 37°C and 5 % CO_2_ and regularly screened to ensure the absence of mycoplasma contamination. Cells were seeded (1.9 × 10^4^ cells/cm^2^ for A549, 3.3 × 10^4^ cells/cm^2^ for SK-N-SH and 1.3 × 10^4^ cells/cm^2^ for U87-MG) 24 hours before treatment.

### Drugs and reagents

Stock solution of paclitaxel (Novasep synthesis) was prepared in dimethyl sulfoxide (DMSO) (Sigma-Aldrich) while vincristine (Lilly, Strasbourg, France) and patupilone (Sigma-Aldrich) were prepared in sterile distilled water. Stock solutions of tiron and potassium cyanide (KCN) were prepared in sterile distilled water, while rotenone was prepared in DMSO. SB216763 was purchased from Sigma-Aldrich. All these solutions were freshly diluted in the culture medium for experiments.

### Cytotoxicity test

Cells were seeded in 96-well plates to be treated during 72 hours with MTAs. Cell survival was measured by using the colorimetric MTT assay (Sigma-Aldrich) as we previously performed [14, 32].

### Measurement of Reactive Oxygen Species

Cells were treated with MTAs, antioxidants, or their simultaneous combination for 6 hours. KCN and rotenone, specific inhibitors of complex I and IV of the mitochondrial respiratory chain were used at 100µM and 100nM respectively, and the O_2_^.-^ scavenger tiron at 2mM. Hydrogen peroxide production was evaluated by H_2_-DCF-DA fluorescence (Life Technologies, Carlsbad, USA) in 96-well black plates. Formation of DCF fluorescent product was measured in a Fluoroskan Ascen FL plate reader as previously described [4]. Superoxide ion generation was measured by WST-1 colorimetric test (Roche, Mannheim, Germany). After 30min of WST-1 incubation (500µM), absorbance was measured at 450nm with a Multiskan plate reader (Ascent). For each assay, the reading was corrected for the estimated cell number: cells were fixed with 1 % glutaraldehyde (Sigma–Aldrich) and stained with 1 % Crystal-violet (Sigma–Aldrich) solution in 20 % Methanol (Sigma–Aldrich).

### Indirect immunofluorescence analysis

Cells were grown on 8-well chamber slides (Labtek, Thermo Scientific, Roskilde, Denmark), precoated for 1 hour with fibronectin (10 μg/ml) for U87-MG or with type I collagen (30µg/ml) for SK-N-SH (Sigma Aldrich), to be treated for 6 hours with MTAs and inhibitors. As previously described [32], cells were incubated with the anti-EB1 (clone 5; BD Biosciences, San Jose, CA) and α-tubulin (clone DM1A; Sigma Aldrich) primary antibodies, and then with Alexa488 or 568-conjugated secondary antibodies (Molecular Probes). Staining was observed using either a Leica DM-IRBE microscope or a Leica TCS SP5 confocal laser-scanning microscope (Leica, Heidelberg, Germany). Images were acquired using Metamoph software or the Leica Confocal software, and were processed using Image J software. For each experimental condition, at least 400 EB1 comets (in 40 cells) were examined to measure their length.

### Western blot analysis

Cells were lysed after 6 hours treatment in RIPA buffer (Tris-HCl 50mM pH 8.0, NaCl 250mM, Triton-X100 0.1 %) with a cocktail of proteases and phosphatases inhibitors (Sigma-Aldrich) added freshly. Protein concentrations were determined using the Bio-Rad Protein Assay (Bio-Rad laboratories, France). Proteins were separated by SDS-PAGE and electrotransferred onto a nitrocellulose membrane. Primary antibodies used were directed against EB1 (clone 5; BD Biosciences), α-tubulin (clone DM1A, Sigma Aldrich), Ser 473 phospho-Akt, total Akt, Ser 9 phospho-GSK3β (Cell Signaling, Boston, USA), total GSK3β (Life Technologies). Peroxydase-conjugated secondary antibodies (Jackson Immunoresearch, Baltimore, USA) and chemiluminescence detection kit (Millipore) were used for visualization, and signal quantification was done with Image J software.

### EB1 immunoprecipitation

Cells were lysed in modified RIPA buffer (Tris-HCl 50mM pH 7.5, NaCl 150mM, 1mM EDTA, 2.5mM MgCl_2_) with a cocktail of proteases and phosphatases inhibitors added freshly. Prior to immunoprecipitation, 5µg of anti-EB1 antibody (clone 5; BD Biosciences) was added to 50µl of protein G-Sepharose beads (Sigma-Aldrich) for 1 hour. Then, an equal amount of each protein lysate was incubated with the previous mixture and rotated overnight at 4°C. After centrifugation, the supernatant was removed and the immune complex was analyzed by Western blot with primary antibodies against EB1 (clone 5; BD Biosciences) and serine, threonine and tyrosine phosphorylated proteins (clone SPM101; Abcam).

### Proliferation test

Cells were grown in 96-well plates, and were fixed at different times with 1 % glutaraldehyde and stained with a 1 % Crystal-violet solution in 20 % Methanol. The stain was eluted with DMSO and absorbance was measured at 600nm with a Multiskan Ascent plate reader.

### Annexin V-FITC/PI staining assay

Following a 24 and 48 hours treatment, cells were exposed to Apoptosis/Necrosis Detection Kit (BD Biosciences) used according to the manufacturer's instructions. The fluorescence was analyzed by flow cytometry analysis (FacSort of Becton Dickinson Immunocytometry Systems, Inc., San Jose, CA) as previously described [67].

### Transwell migration assay

Cells were allowed to migrate for 6 hours in a transwell migration chamber (0.8 μm filter, BD Bioscience); for details see our previous work [32]. Pictures of the lower sides of filters were taken with an Olympus microscope. Six fields per condition were imaged and transmigrated cells were counted. Results were expressed as a percentage of transmigrated cells compared with no treatment condition.

### Plasmid constructs and transfection

pEB1-T166 and S155 were derived from pEB1-GFP (human EB1 in pEGFP N1 to express EB1-GFP) by mutation of the T166 or/and S155 of EB1 using the Quickchange II site directed mutagenesis kit (Agilent Technologies, Les Ulis, France) according to the manufacturer's protocol. The T166 and S155 were both replaced by an A by mutation. The primers (mutations are underlined) used were:

T166 site (561 bp from origin):

MuEB1T166 F 5' GAGGCCCATCTCAGCACAGAGAACC 3'

MuEB1T166 R 5' GGTTCTCTGTGCTGAGATGGGCCTC 3'

S155 site (527 bp from origin):

MuEB1S155 F 5'CCTCTCACTGCTAGCAGTGCAGCTC 3'

MuEB1S155 R 5' GAGCTGCACTGCTAGCAGTGAGAGG 3'

The resulting mutant cDNA (pEB1–T166A and pEB1–S155A) constructs were sequenced to confirm their integrity before use (Beckman Coulter Genomics). U87-MG cells were then transiently transfected with 3µg pEB1, pEB1–T166A or pEB1–S155A using Lipofectamine 2000 reagent (Life Technologies) according to the manufacturer's instructions. To establish stable clones, cells were selected with 0.8 µg/ml geneticin (Life Technologies).

### Fluorescent time-lapse video microscopy

Time-lapse acquisitions for microtubule dynamics experiments (*wt*EB1-GFP, T166A-GFP and S155A-GFP U87-MG cells) were performed with a Leica DM-IRBE equipped with a 60X / NA 1.49 objective lens. Thirty-one images per cell were acquired at 2-s intervals using a digital camera (CCD camera Coolsnap FX; Princeton Instruments). Analysis of microtubule dynamics was performed using the manual tracking personalized plug-in for Image J software. Position of EB1 comets was detected by thresholding a filtered image and the centroids of individual comets were followed over time. Changes in length exceeding 0.067 µm were considered as growth events. Catastrophe frequency was calculated for each individual comet tracked, and corresponds to the inverse of the total length of growth. Decoration time was calculated by dividing EB1 comet length by microtubule growth rate. At least 30 microtubules were analyzed for each experimental condition.

### Statistical analysis

Each experiment was performed at least in triplicate. Data are presented as mean ± S.E.M. Statistical significance was tested using Student's t test. A significant difference between two conditions was recorded for *, *p*<0.05; **, *p*<0.01; ***, *p*<0.001.

## SUPPLEMENTARY INFORMATION AND FIGURES








