# Supplementary material for: ROS-mediated EB1 phosphorylation through Akt/GSK3β pathway: implication in cancer cell response to microtubule-targeting agents
Source: Oncotarget. 2014 May 18;5(10):3408–23. doi: 10.18632/oncotarget.1982 (PMC4102819; doi:10.18632/oncotarget.1982)
Supplement: Supplementary file 4 [file oncotarget-05-3408-s004.pdf]

ROS-mediated EB1 phosphorylation through Akt/GSK3β pathway: implication in cancer cell response to Microtubule-Targeting Agents

Supplementary Material

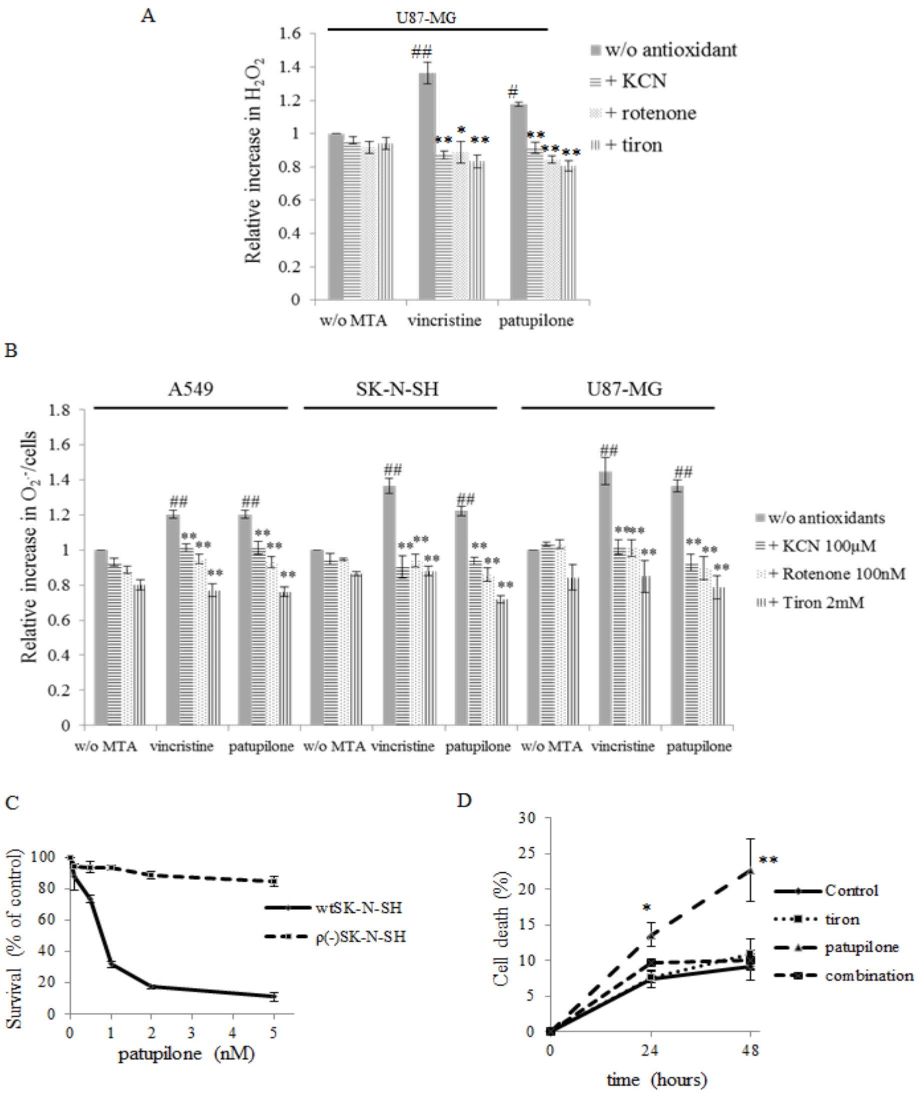

Supplementary Figure 1: (A) Relative generation of hydrogen peroxide by the H_2DCFDA fluorescence test in U87-MG cells incubated with 2 nM of vincristine or patupilone, ROS inhibitors/scavengers or their combination for 6 h (MTAs are compared to control while combinations are compared to MTAs alone). (B) Relative production of superoxides by WST-1 assay in A549, SK-N-SH and U87-MG cells exposed to 2 nM of vincristine or patupilone, ROS

inhibitors/scavengers or their combination for 6 h (MTAs are compared to control while combinations are compared to MTAs alone). (C) *wt*SK-N-SH and $\rho^{(-)}$SK-N-SH cells survival revealed by the MTT test after a 72 h-exposition to patupilone. (D) Detection of apoptosis by flow cytometry analysis of annexin-V and propidium iodide staining in A549 cells treated with patupilone (2nM), tiron or their combination for 24 and 48 h. Data are presented as mean ± S.E.M. Student's *t*-test *, $p<0.05$; **, $p<0.01$.

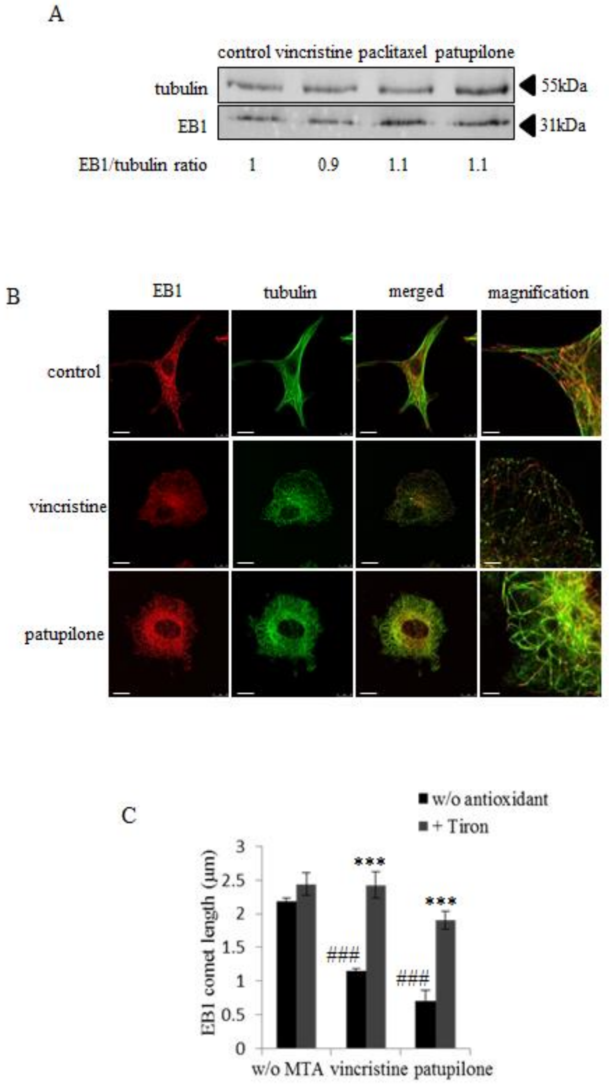

Supplementary Figure 2: (A) Western Blot analysis of EB1 expression under 6 h-treatment with 2nM vincristine, paclitaxel or patupilone in A549 cells. Ratio EB1/tubulin relative of control; α–tubulin was used as loading control. (B) Double indirect immunofluorescence staining of EB1 (red) and α–tubulin (green) in U87-MG cells incubated with 2 nM of patupilone or vincristine for 6 h. Scale bars, 20µm. Magnified images: Scale bars, 5µm. (C) Quantification of EB1 comet length in U87-MG cells under 6 h-treatment of vincristine or patupilone, tiron or their combination (MTAs conditions are compared to control while combinations are compared to MTAs alone). Data are presented as mean ± S.E.M. Student's *t*-test *, $p<0.05$; **, $p<0.01$.

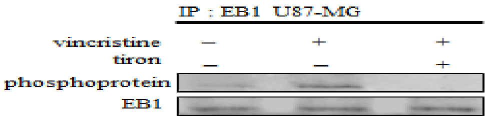

Supplementary Figure 3: (A) U87-MG cells were treated for 6 h with 2nM of vincristine or combined with tiron, and lysed. Immunoprecipitation was performed as described in legend of Figure 3A.

A

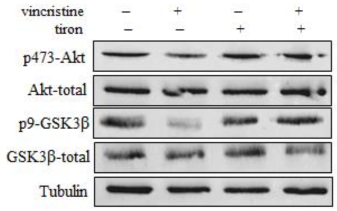

B

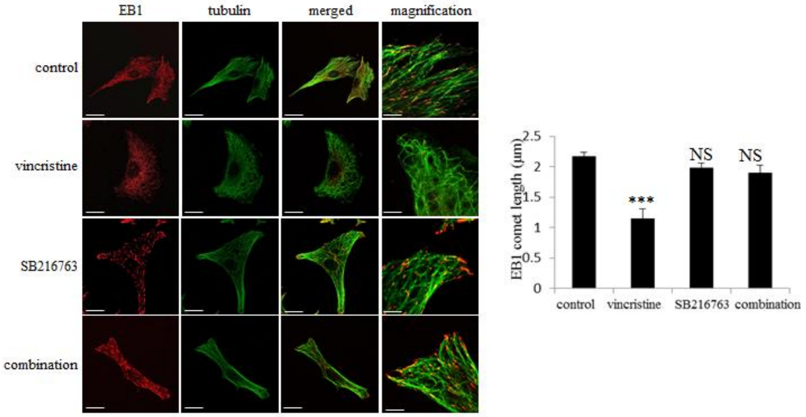

Supplementary Figure 4: (A) Western blot analysis of expression and activity of Akt and GSK3β under 6 h-treatment with 2nM of vincristine, tiron or their combination in A549 cells. (B) Double indirect immunofluorescence staining of EB1 (red) and α–tubulin (green) and quantification of EB1 comet length in U87-MG cells incubated with 2nM of vincristine, SB216763 or their combination for 6 h. Scale bars, 20μm. Magnified images: Scale bars, 5μm. Data are presented as mean ± S.E.M. Student's *t*-test *, $p<0.05$; **, $p<0.01$.
